# Aquatic Therapy Versus Land-Based Therapy in Patients with Parkinson’s Disease: A Systematic Review

**DOI:** 10.3390/jfmk10020170

**Published:** 2025-05-12

**Authors:** Gema Santamaría, Mario Fernández-Gorgojo, Eduardo Gutiérrez-Abejón, Blanca García Gómez, Ángela Molina, Diego Fernández-Lázaro

**Affiliations:** 1 Department of Anatomy and Radiology, Faculty of Health Sciences, Campus of Soria, University of Valladolid, 42003 Soria, Spain; gema.santamaria@uva.es; 2 Faculty of Health Sciences, Campus of Ponferrada, University of León, 24416 Ponferrada, Spain; mafeg@unileon.es; 3 Pharmacological Big Data Laboratory, Department of Cell Biology, Genetics, Histology and Pharmacology, Faculty of Medicine, University of Valladolid, 47003 Valladolid, Spain; egutierreza@saludcastillayleon.es; 4 BioCritic, Group for Biomedical Research in Critical Care Medicine, 47003 Valladolid, Spain; 5 Centro de Investigación Biomédica en Red de Enfermedades Infecciosas (CIBERINFEC), Instituto de Salud Carlos III, 28029 Madrid, Spain; 6 Pharmacy Directorate, Castilla y León Health Council, 47007 Valladolid, Spain; 7 Valladolid Este Primary Care Department, 47010 Valladolid, Spain; 8 Department of Business Organization and Marketing and Market Research, Faculty of Business and Labor Sciences, University of Valladolid, Campus of Soria, 42004 Soria, Spain; blanca.garcia.gomez@uva.es; 9 Faculty of Chemistry, Rovira and Virgili University, 43007 Tarragona, Spain; angela.molina@estudiants.urv.cat; 10 Area of Histology and Neurobiology Research Group, Faculty of Medicine, University of Valladolid, 47003 Valladolid, Spain

**Keywords:** Parkinson’s disease, aquatic therapy, land-based therapy, quality of life, balance

## Abstract

**Background:** Parkinson’s Disease (PD) is the second most prevalent neurodegenerative disease worldwide. Motor and non-motor symptoms of PD cause functional disabilities. Aquatic-based therapeutic exercise (AT) is a potential approach that may improve the management of PD, given its hydrostatic and hydrodynamic properties. We aimed to evaluate the effectiveness and safety of AT compared to traditional land-based therapeutic exercise (LT) in patients with PD. **Methods:** Based on the Preferred Reporting Items for Systematic Review and Meta-Analysis (PRISMA) guidelines, we systematically reviewed studies indexed in PubMed, Scopus, Web of Science, PEDro, CINAHL, and Cochrane. Registered in PROSPERO (CRD42024528310), this review involved original studies published from 2014 to December 2024, with a randomized controlled trial (RCT) design, in which the intervention group performed AT, and the control group performed LT. The outcomes evaluated were balance, gait, quality of life, strength, mental health, pain, flexibility, and sleep quality. **Results:** Of the 413 records identified, 135 duplicates were removed, and 265 did not meet the selection criteria. Thirteen RCTs comprising 511 patients (age range: 50–80 years) were eligible. Most studies reported beneficial effects of AT, with no serious adverse events. Compared to LT, AT led to significant improvements (*p* < 0.05) in quality of life, mental health, pain, flexibility, and sleep quality. No evidence was provided of the beneficial effects of AT compared to LT on balance, gait, and strength; however, significant improvements were observed in the AT group from baseline (*p* < 0.05). **Conclusions:** AT appears to be a safe and effective intervention for improving the quality of life, mental health, pain, flexibility, and sleep quality in PD patients. While balance, gait, and strength may also benefit, the evidence comparing AT to LT remains inconclusive due to variability in study protocols.

## 1. Introduction

Parkinson’s disease (PD) is a multisystem neurodegenerative disease characterized by deposits of α-synuclein in multiple regions of the nervous system [1,2]. The main regions are the substantia nigra, nucleus basalis of Meynert, and dorsal motor nucleus of the vagus nerve [2,3]. Patients with PD exhibit motor symptoms such as resting tremors, bradykinesia, stiffness, postural instability, and postural and gait alterations [1,2,4]. Non-motor symptoms, although often underestimated, can be equally disruptive motor symptoms, severely affecting patients’ quality of life (QoL) and autonomy [4]. These include fatigue, sleep disturbance, depression, anxiety, cognitive impairment, dementia, hallucinations, urinary problems, and sexual dysfunction [2,3,4,5]. PD has become one of the main causes of disability worldwide, being the second most prevalent neurodegenerative disease after Alzheimer’s Disease [6]. The prevalence of PD in industrialized countries is estimated to be 0.3%, affecting up to 3% of the population over 80 years old [2]. PD is experiencing an alarming global increase, from 2.5 million cases in 1990 to 6.1 million in 2016, and these rates are expected to double by 2030 [1,3]. This high increase has led some researchers to characterize PD as a pandemic due to its wide geographical spread and exponential growth [7,8]. PD has a considerable impact on public health and the global economy. The United States spent USD 14.4 billion in 2010 on PD treatment, equivalent to USD 22,800 per patient [9]. For all these reasons, PD has become a major public health problem requiring an urgent solution.

Despite advances in the understanding of PD, there is currently no treatment able to stop or reverse the neurodegenerative process [1]. Conventional treatment is focused on symptom control, mainly through the administration of levodopa in combination with carbidopa [1,2]. Although this drug therapy has increased survival from 9 to 13 years [2], side effects such as fatigue, dyskinesias, anxiety, and somnolence are common and can complicate the management of the disease [4]. In order to reduce doses and modulate side effects, therapeutic exercise has been proposed as a possible adjuvant [10,11,12,13,14]. In particular, aquatic therapy (AT) has emerged as an alternative therapeutic option due to water properties like floatability, hydrostatic pressure, viscosity, and thermodynamics [15,16]. AT is considered a non-invasive, non-pharmacological treatment for chronic diseases [17,18]. AT has been shown to significantly improve balance, gait, strength, and QoL in patients with PD [19,20,21]. However, there is no consensus on the effectiveness of AT compared to land-based therapeutic exercise (LT). Some authors argue that AT provides greater benefits than LT [20,22,23], while others recognize its benefits but do not consider AT superior to LT [19,24]. The effectiveness of AT in PD has been the subject of several reviews in the scientific literature [20,21,23,24,25,26]. However, these reviews have highlighted substantial limitations, including small sample size [21,24], inclusion of uncontrolled clinical trials [25], and control group heterogeneity [20,23,26]. These methodological shortcomings underline the need for a rigorous systematic review that addresses the current evidence and provides a critical assessment of the effectiveness of AT compared to LT. In this context, the aim of the present study was to systematically review the available scientific evidence on the effectiveness of AT compared to LT in patients with PD, analyzing aspects such as QoL, balance, gait, and strength.

## 2. Materials and Methods

The present systematic review was conducted and reported following the Preferred Reporting Items for Systematic Reviews and Meta-analyses (PRISMA) guidelines [27] (Appendix A). The systematic review protocol was registered in PROSPERO (CRD42024528310).

### 2.1. Search Strategy

The PICO question model was used according to the recommendations of Evidence-Based Medicine [28]: P (population): men and women diagnosed with PD classified within stages 1–3 on the Hoehn and Yahr Scale (H&Y), excluding patients with dementia, cognitive impairments, cardiac pathologies, or other associated conditions. I (intervention): aquatic-based therapeutic exercise. C (comparison): land-based therapy. O (outcomes): balance, gait, QoL, strength, pain, flexibility, mental health, and sleep quality. S (study design): randomized clinical trial. For article selection, a structured search was carried out using the electronic databases Medline (PubMed), Scopus, Web of Science, Physiotherapy Evidence Database (PEDro), CINAHL, and Cochrane between October and December 2024. The search strategy, detailed in Appendix B, contained a combination of Medical Subject Headings (MeSH) and free words such as “Parkinson Disease”, “Parkinson”, “Parinson’s Disease”, “Aquatic Therapy”, “Aquatic Exercise Therapy”, “Water Exercise Therapy”, and “Ai Chi Therapy” linked by the Boolean operators “AND” and “OR”. Two authors independently performed the search for published studies, and a third reviewer participated in case of disagreement. In addition, the bibliographic references of all included articles and some of the excluded articles were reviewed, and ResearchGate was checked in order to identify relevant titles that might have been missed by the search strategy.

### 2.2. Selection Criteria

The following inclusion criteria were established for the studies selection: (1) Patients with PD (excluding studies whose population had other associated pathologies); (2) Intervention group (IG) treated with AT; (3) Control group (CG) treated with LT; (4) Randomized clinical trials that report primary or secondary outcomes on QoL, balance, gait, strength, flexibility, pain, sleep quality, or mental health; (5) Score equal to or greater than 6 on the Critical Appraisal Skills Program in Spanish (CASPe) questionnaire [29]; (6) Published in Spanish, English, Italian and Portuguese; (7) Published from 2014 onwards.

All studies that did not meet these criteria were excluded.

### 2.3. Data Extraction

The first author’s surname, year of publication, country of publication, sample size, gender, age, intervention of IG and CG, measurement scales used, and final results were extracted from all included studies. The data extraction process was independently carried out by two researchers using a spreadsheet (Microsoft Inc., Seattle, WA, USA). In case of disagreements, a third reviewer was involved in this process.

### 2.4. Assessment of Methodological Quality and Risk of Bias

The CASPe questionnaire [29] was used to assess the methodological quality of the included studies. Additionally, the risk of bias was assessed using the Cochrane risk of bias tool [30].

## 3. Results

### 3.1. Study Selection

**Figure 1:**
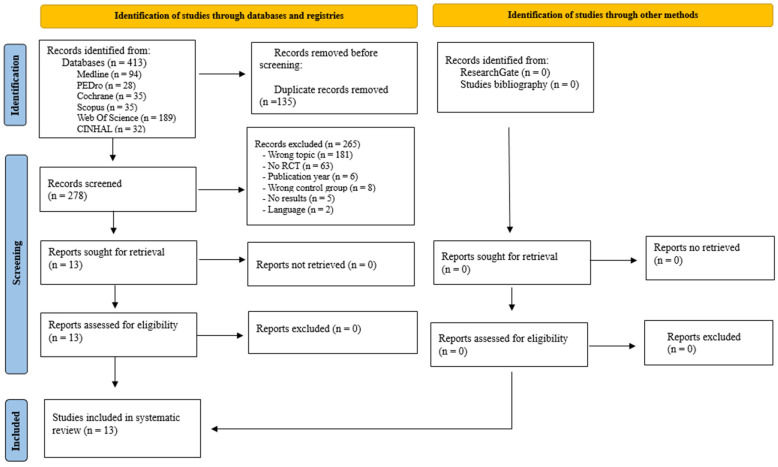
Flow chart of study selection for the literature review (PRISMA) [27].

The literature search resulted in a total of 413 records from Medline (*n* = 94), PEDro (*n* = 28), Cochrane (*n* = 35), Scopus (*n* = 35), Web of Science (*n* = 189), and CINAHL (*n* = 32). No eligible studies were identified in ResearchGate or the reference list of relevant studies. After duplicate removal (*n* = 135), the titles and abstracts of the remaining 278 publications were analyzed. A total of 265 publications were eliminated for not being related to the topic of interest (*n* = 181), not being randomized clinical trials (*n* = 63), having been published prior to 2014 (*n* = 6), having a CG who did not perform LT (*n* = 8), being protocols without results (*n* = 5), and being in a language other than English, Spanish, Italian, and Portuguese (*n* = 2). The remaining 13 trials were examined in full text and met the selection criteria for inclusion in the systematic review [31,32,33,34,35,36,37,38,39,40,41,42,43] (Figure 1).

### 3.2. Assessment of Methodological Quality

All included articles met the minimum requirements for methodological quality, scoring above 6 on the CASPe questionnaire [31,32,33,34,35,36,37,38,39,40,41,42,43]. Scores ranged from 8 [37,40] to 10 points [32,36,38,39]. Due to the type of intervention, none of the trials met the criterion of complete blinding. All therapists and participants knew the group to which they had been assigned [31,32,33,34,35,36,37,38,39,40,41,42,43]. However, in nine studies, the assessors were blinded [31,33,34,35,37,38,40,41,43]. The effect sizes were large (d = 0.8) [31,32,37,39,40,43], medium (0.5 < d < 0.8) [36,38,42], and small (d < 0.5) [33,34,35,41] (Table 1).

### 3.3. Characteristics of Participants and Interventions

The total initial number of volunteers was 511, of which 464 completed this study, representing a dropout rate of 9.19% [31,32,33,34,35,36,37,38,39,40,41,42,43]. A total of 64% of the participants were men, and 36% were women between the ages of 50 and 80 years [31,32,33,34,35,36,37,38,39,40,41,42,43]. All studies included men and women in their samples [31,32,33,34,35,37,38,39,40,41,42,43], except Shahmohammadi et al. [36], who only included men. Participants had PD stages 1–3 on the H&Y scale [33,34,35,37,38,40,41,42], 2–3 H&Y [31,32,36], and 2.5–3 H&Y [39,43]. The investigation was conducted during ON [31,32,36,37,38,39,40,41,42,43] or OFF [33,34,35] periods of medication (Table 2).

Table 3 shows the characteristics of the interventions. Ten studies proposed the same protocol duration and frequency, duration, and intensity of the sessions for the IG and the CG, differing only in the type of exercise performed [32,33,34,35,36,37,38,39,41,42]. In the remaining three studies [31,40,43], the IG performed the same LT program as the CG in addition to AT. The duration of the protocols varied from 4 [31,43] to 12 weeks [37,41,42]. Each week, one [37], two [33,34,35,40,41,42], three [31,36,43], or five [32,38,39] sessions were performed. The duration of the session ranged from 30 [40] to 60 min [31,32,36,37,38,39,41,42,43]. All sessions were supervised by a qualified professional [31,32,33,34,35,36,37,38,39,40,41,42,43]. The techniques used in the main part were Ai Chi [32,33,34,35], WATSU [40], Halliwick [37], gait work [31,36,41,42], balance [37,38,39,41,42,43], proprioception [31,43], coordination [41,43], strength work [37,39,41,42], aerobic work [37], and joint mobility [37,42,43].

### 3.4. Evaluation of the Results

**Figure 2:**
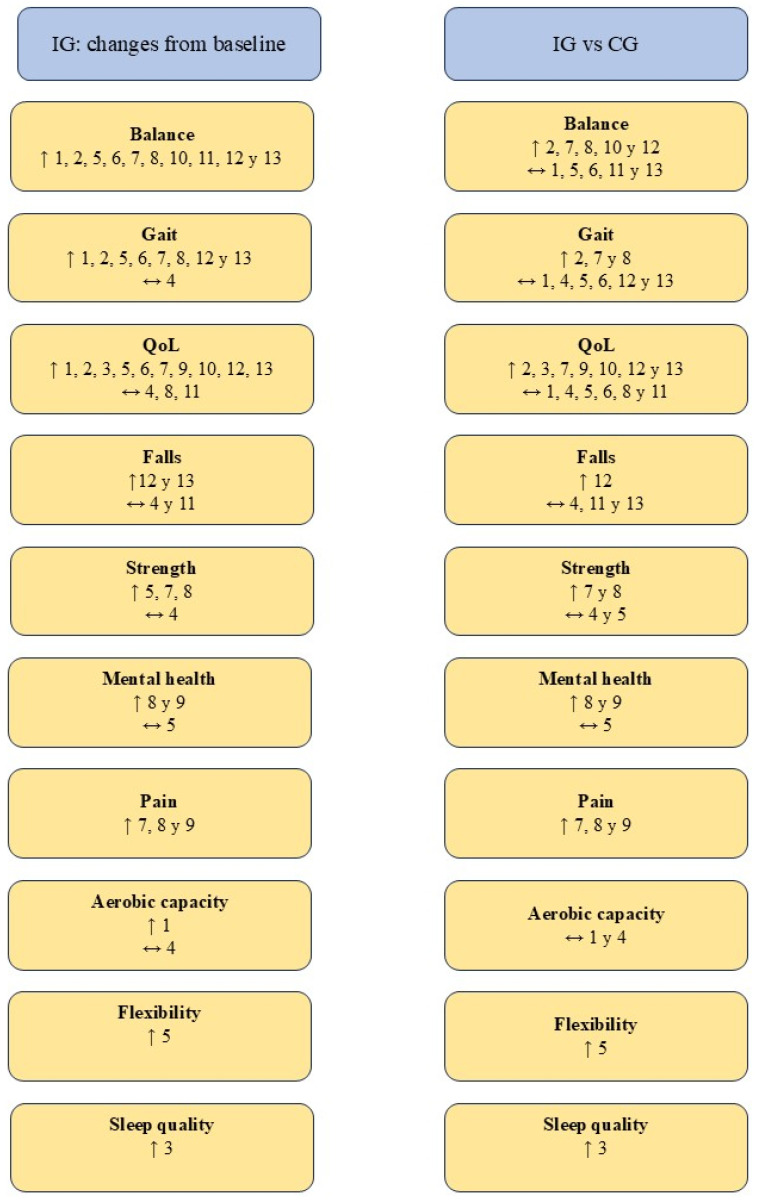
Summary of the results obtained. Abbreviations. ↑ Statistically significant improvements (*p* < 0.05); ↔ No statistically significant difference (*p* > 0.05); CG: control group; IG: intervention group; QoL: Quality of life. Authors. 1. Clerici et al. [31], 2. Kurt et al. [32], 3. Loureiro et al. [40], 4. Nogueira et al. [41], 5. Nowak [42], 6. Palamara et al. [43], 7. Pérez de la Cruz [35], 8. Pérez de la Cruz [34], 9. Pérez de la Cruz [33], 10. Shahmohammadi et al. [36], 11. Terrens et al. [37], 12. Volpe et al. [39], 13. Volpe et al. [38].

The parameters assessed and the results obtained after the interventions are presented in Table 2. In addition, the main findings are summarized in Figure 2.

#### 3.4.1. Balance

Changes in balance were assessed by 10 of the studies included in this systematic review [31,32,34,35,36,37,38,39,42,43]. The 10 trials found statistically significant (*p* < 0.05) improvements in IG balance from baseline [31,32,34,35,36,37,38,39,42,43]. However, only five of them reported statistically significant (*p* < 0.05) improvements over CG [32,34,35,36,39], while the remaining five did not find any difference (*p* > 0.05) [31,37,38,42,43].

#### 3.4.2. Gait

Nine of the 13 included studies evaluated the effects of AT on gait [31,32,34,35,38,39,41,42,43]. All studies, with the exception of Nogueira et al. [41], reported statistically significant (*p* < 0.05) improvements in IG gait from baseline [31,32,34,35,38,39,42,43]. A greater disparity has been observed in relation to changes in IG compared to CG. Three studies reported significant improvements (*p* < 0.05) [32,34,35] in contrast to the remaining six studies, which showed no differences between groups (*p* > 0.05) [31,38,39,41,42,43].

#### 3.4.3. Quality of Life

QoL related to PD symptomatology has been the most studied parameter, assessed by all 13 studies [31,32,33,34,35,36,37,38,39,40,41,42,43]. Ten of the studies recorded statistically significant (*p* < 0.05) increases in the IG compared to baseline [31,32,33,35,36,38,39,40,42,43]. However, only seven of them found better results in IG (*p* < 0.05) than in CG [32,33,35,36,38,39,40], while the remaining six did not show significant differences (*p* > 0.05) [31,34,37,41,42,43].

#### 3.4.4. Strength

Lower limb strength was assessed by four trials using the Sit-to-stand test [34,35,41,42]. Three of them found statistically significant (*p* < 0.05) increases in IG over baseline [34,35,42], but only Pérez de la Cruz [34,35] reported statistically significant (*p* < 0.05) increases over CG. In a complementary way, Nowak [42] assessed the isometric strength of the knee and ankle musculature and the isokinetic strength of the knee. In none of these parameters, IG was superior to CG (*p* > 0.05) despite significant increases (*p* < 0.05) compared to baseline [42].

In addition, Nogueira et al. [41] and Nowak [42] assessed upper limb strength with manual dynamometry. The results of the two studies indicate that there are no significant differences (*p* > 0.05) between groups [41,42].

#### 3.4.5. Other Parameters Evaluated

The effect of AT on mental health was evaluated by three studies [33,34,42]. Pérez de la Cruz [33,34] found in his two studies statistically significant (*p* < 0.05) improvements in IG compared to CG, while Nowak [42] did not find differences in IG compared to CG or baseline. Pain was studied by Pérez de la Cruz in his three investigations [33,34,35]. This author reported a statistically significant (*p* < 0.05) reduction in pain in IG relative to CG and baseline in all three studies [33,34,35]. Aerobic capacity was assessed by Clerici et al. [31] and Nogueira et al. [41], neither of whom found significant differences between groups (*p* > 0.05). Lower limb flexibility was studied by Nowak [42], who reported significant increases (*p* < 0.05) in the IG with respect to the CG and baseline. Finally, Loureiro et al. [40] evaluated the effect of TA on sleep quality, finding a significant increase (*p* < 0.05) in IG compared to CG and baseline.

### 3.5. Bias Assessment

**Figure 3:**
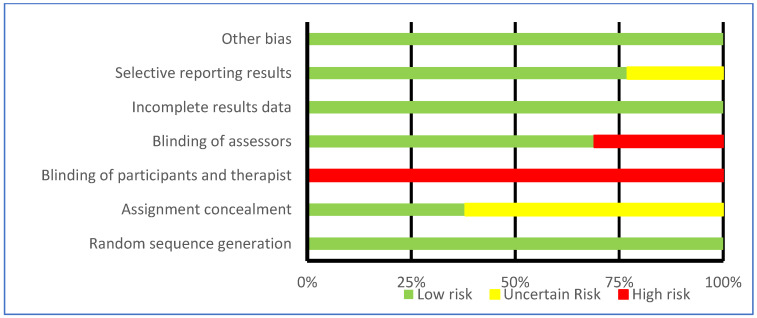
Results of risk of bias assessment of included studies—Cochrane tool [30].

The assessment of bias is represented in Table 4 and Figure 3 according to Cochrane recommendations [30]. All studies showed low risk in random allocation of participants, incomplete outcomes data, and other biases [31,32,33,34,35,36,37,38,39,40,41,42,43]. In contrast, all studies were at high risk of blinding participants and therapists [31,32,33,34,35,36,37,38,39,40,41,42,43]. Eight of the studies did not specify in the methodology how allocation concealment was performed [32,33,34,35,36,39,41,42]. The three studies by Pérez de la Cruz [33,34,35] had an unclear risk of selective reporting of results.

## 4. Discussion

Overall, AT has been demonstrated to be an effective therapeutic approach in improving balance, gait, QoL, strength, pain, mental health, flexibility, and sleep quality. In addition, participants who practiced AT showed significantly (*p* < 0.05) higher improvements in QoL, mental health, flexibility, sleep quality, and pain compared to LT. However, there is no evidence of superior effects of AT on balance, gait, and strength. It is important to note that none of the 13 clinical trials reviewed reported that LT was superior to AT in any of the parameters assessed. Therefore, AT has been shown to be a superior or at least equal alternative to LT in the treatment of PD. No adverse effects of AT practice have been reported in PD patients, demonstrating that AT is a safe therapy [31,32,33,34,35,36,37,38,39,40,41,42,43]. To ensure safety, all interventions were supervised by a qualified physiotherapist following Australian Physiotherapy Association guidelines [44].

### 4.1. Balance

Balance disturbances and falls are among the motor symptoms that most concern PD patients, second only to tremors [45]. As PD progresses, the processing of vestibular, visual, and proprioceptive signals responsible for maintaining balance becomes impaired [46]. In this context, all 10 clinical trials assessing balance reported statistically significant (*p* < 0.05) increases after AT [31,32,34,35,36,37,38,39,42,43]. The aquatic environment may compensate for the altered proprioceptive signal processing by acting on peripheral sensory receptors [47]. Increased proprioceptive input may contribute to improved balance and body alignment [47]. In addition, AT, due to the floatability and viscosity of water, helps to organize the information and response of cortical regions, providing coordinated motor strategies and improving balance [21]. Finally, in-water training reduces the fear of falling and allows more challenging balance exercises to be performed for longer periods of time [48].

There is no consensus on the effectiveness of AT compared to LT on balance. Five trials have reported that AT was superior to LT [32,34,35,36,39], while the remaining five have found no significant difference between groups [31,37,38,42,43]. The only difference in the protocols used seems to be in water temperature. Authors who found superior AT kept the water at 30–32 °C [32,34,35,36,39], while authors who did not find a difference maintained it at 34–34.7 °C [31,37] or did not specify it [38,42,43]. Water temperature is an important factor to consider. It has been suggested that warm water may stimulate skin thermoreceptors and increase the activity of cortical sensory and motor areas, promoting sensory-motor integration [23]. It would, therefore, have a positive effect on balance. However, it should be noted that warm water also reduces muscle tone. A plausible hypothesis is that excessively hot water drastically reduces the tone of the musculature responsible for maintaining balance, impairing the correct performance of its function. A final point to highlight is that neither of the two studies that added AT to LT found improvements with respect to CG [31,43]. This suggests that practice AT does not provide additional benefits when LT is already being performed.

### 4.2. Gait

Following AT intervention, gait improved significantly (*p* < 0.05) in eight [31,32,34,35,38,39,42,43] of the nine studies that assessed it [31,32,34,35,38,39,41,42,43]. Gait improvement has been reported to be closely related to the balance benefits mentioned above [49]. Increasing postural stability allows patients to focus on walking correctly, which reduces the freezing of gait episodes [49]. In addition, walking underwater can positively influence motor learning, allowing patients to adapt to environmental perturbations [50]. AT achieves changes in spatiotemporal parameters and lower limb kinematics, generating clinically significant effects [50]. On the other hand, AT was only superior to LT in trials that used Ai Chi [32,34,35], indicating that it is the most effective methodology in improving gait. A previous systematic review reported that Ai Chi is effective in improving balance, pain, and functional mobility in healthy adults and patients with neurological diseases [48]. This is consistent with the results found in this review, especially in relation to gait. Many of the Ai Chi benefits are thought to come from conscious movement, giving it superior effects to LT and other types of AT [48].

Freezing of gait, despite being a very limiting phenomenon and a frequent cause of falls [51], has only been evaluated by one study [31]. Clerici et al. [31] reported that AT significantly (*p* < 0.05) reduces freezing of gait but did not find a difference with LT. In their protocol, Clerici et al. [31] added AT to LT, which might indicate that AT does not bring additional benefits when LT is already performed, as in the case of balance. Considering that the freezing of gait is caused by a loss of sensory information at the central level [52], AT could be expected to reduce the freezing of gait by increasing sensory information [47]. However, future studies are needed to clarify the effectiveness of AT alone on freezing gait.

### 4.3. Quality of Life

Participants’ QoL improved significantly (*p* < 0.05) after AT practice [31,32,33,35,36,38,39,40,42,43]. Seven studies have found that TA was superior (*p* < 0.05) to LT in improving QoL [32,33,35,36,38,39,40], while six studies did not report a difference (*p* > 0.05) [31,33,37,41,42,43]. Similarly, a recent systematic review by Gomes-Neto et al. [53] concluded that AT provides greater benefits than LT on QoL. AT could increase the QoL of PD patients for the following four reasons: (1) The improvements in balance and gait discussed in the previous points. (2) AT achieves mental health benefits superior to LT [33,34]. It has been reported that AT can increase self-efficacy, improve mood and self-esteem, relieve stress, and reduce anxiety and depression, helping to increase QoL [54,55,56]. (3) AT results in superior improvements in sleep quality than LT [40]. Given that sleep disorders negatively affect QoL and cognitive status, their correction could have a positive impact on QoL [57,58]. (4) AT reduces pain significantly more than LT [33,34,35]. Pain sensitivity is increased in PD due to abnormal temporal summation and impaired central pain processing, with lower pain thresholds [23]. However, pain is underestimated, underdiagnosed, and undertreated in PD [48]. Hot water immersion has anti-allodynic effects mediated by peripheral opioid, cannabinoid, and adenosine receptors in animal models [59]. This makes AT an effective option for pain management in PD by increasing QoL.

### 4.4. Strength and Flexibility

Strength was assessed by four studies [34,35,41,42]. Three of them found significant (*p* < 0.05) increases over baseline [34,35,42], but only two over CG [34,35]. Analyzing the trial protocols, the pattern identified above is repeated, with only Ai Chi achieving greater increases in strength than LT [34,35]. Ai Chi is not only effective in increasing strength in PD, but it has also been reported to provide benefits in strength in other diseases, such as multiple sclerosis [60]. Changes in strength after performing an AT protocol may be due to an increase in resistance to movement produced by water viscosity. This requires a greater involvement of the muscle strength components during exercise [61].

The impact of AT on flexibility was only evaluated by Nowak [42], who found significant (*p* < 0.05) increases over LT. This may be attributed to the fact that immersion in warm water increases the extensibility of collagen tissue and inhibits stretch reflex excitability, improving flexibility and relieving muscle stiffness [23].

### 4.5. Practical Applications

**Figure 4:**
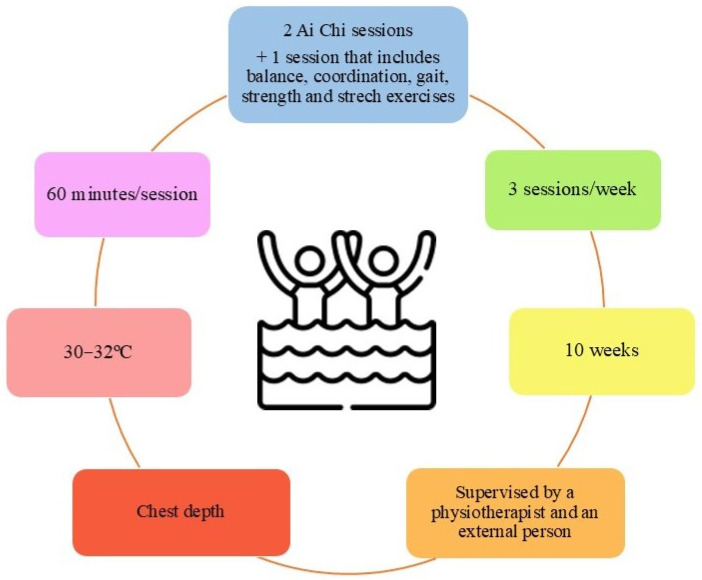
Proposed protocol for aquatic therapy in patients with Parkinson’s Disease.

In order to unify the wide variety of AT protocols designed for PD patients, an evidence-based AT protocol from the 13 trials included in this review is presented in Figure 4.

### 4.6. Reflections on the Role of Aquatic and Land-Based Physical Exercise in Parkinson’s Disease

Strong clinical evidence has shown a positive correlation between physical exercise and the amelioration of symptoms and side effects of Parkinson’s disease. In this context, the question arises as to which approach, aquatic or land-based, may be of most benefit to Parkinson’s sufferers. The underlying molecular mechanisms may shed some light on the results reported and guide sports therapies in this regard.

Irrespective of whether it is aquatic or land-based, physical exercise has shown cognitive improvements in patients with neurodegenerative diseases. Most cognitive benefits associated with exercise have been related to the production of growth factors at local and systemic levels within the hippocampus [62]. Brain-derived neurotrophic factor (BDNF), which is involved in the expansion of neural networks and cognitive–behavioral functions, plays a key role. Notably, a decrease in BNDF has been characterized in the pathological picture of Parkinson’s disease, and the increase in this neurotrophin is, in itself, a potential therapy for the condition [62]. Szuhany et al. [63] review in a meta-analysis of 14 studies how physical exercise leads to a significant increase in BNDF levels. At the brain level, despite the multitude of studies documenting this issue, it is not known how exercise stimulates BDNF production. However, the hemodynamic hypothesis suggests that the elevation of cerebral blood flow by exercise increases the activity of tissue plasminogen activator (tPA), which is responsible for cleaving proBNDF and producing mature BNDF [64].

Wrann et al. [65] demonstrated through their research that FNDC5, an exercise-induced muscle protein initially secreted as irisin, correlates with BNDF expression. FNDC5 is regulated by PGC-1α and has been shown to increase under conditions of hippocampal endurance exercise. The increased expression of FNDC5 in primary cortical neurons results in the upregulation of BNDF expression [65]. Water exercise leads to an increase in BNDF and, consequently, cognitive function [66,67]. Although an increase in BNDF is also found in floor exercises, we suggest that the properties of water may help to achieve higher levels of BNDF, not necessarily due to the type of exercise, but rather because it allows more effort to be exerted without feeling fatigued. This leads to increased performance, which could result in higher levels of BNDF.

An additional beneficial effect of exercise would be on AMP-activated protein kinase (AMPK) activity in skeletal muscle. This has a positive impact on several brain processes, including improved learning and memory abilities, increased neurogenesis, and regulation of genes associated with mitochondrial function in the hippocampus [68]. Aquatic exercise programs reduce chronic low-grade inflammation by decreasing pro-inflammatory markers like tumor necrosis factor α (TNF-α) and increasing anti-inflammatory cytokines such as interleukin 10 (IL-10). This anti-inflammatory response may indirectly enhance pathways like AMPK [68]. The hydrostatic pressure of water may improve circulation and lymphatic drainage, enhancing the systemic anti-inflammatory response. Additionally, the cooling effect of water reduces exercise-induced inflammation, further promoting cytokine balance [69].

Physical activity has also been shown to increase circulating levels of insulin-like growth factor 1 (IGF-1). This hormone seems to contribute to the alleviation of Parkinson’s symptomatology by increasing hippocampal cell supersurvival capacity and being protective against brain injury [66]. It also has the ability to cross the BBB and induce BDNF synthesis in response to exercise [66]. Studies comparing aquatic and non-aquatic exercises found that IGF-1 levels increased significantly in both modalities. However, Aquatic exercise also stimulates vascular endothelial growth factor (VEGF), which works synergistically with IGF-1 to enhance vascularization and cognitive function [66]. This dual effect may be less pronounced in non-aquatic settings due to differences in exercise dynamics. Also, the buoyancy of water reduces joint stress, allowing participants to engage in higher-intensity movements safely, which may enhance the release of growth factors like IGF-1 compared to LT.

It is, therefore, suggested that aquatic exercises may provide unique physiological benefits due to the properties of water (buoyancy, resistance, hydrostatic pressure) that favor and enhance the exercise-associated synthesis of key molecules in cognitive enhancement, neuroprotection, and reduction in inflammation.

### 4.7. Future Lines

During the course of this systematic review, a number of knowledge gaps have been identified and need to be addressed. Firstly, future research should clarify the effectiveness of AT alone on freezing of gait, a symptom that significantly affects PD patients’ mobility. Also, no study has evaluated the effectiveness of AT on transfers nor the effect of Ai Chi on falls. On the other hand, the effect of AT on strength should be further studied, as only four studies evaluated it, leaving a considerable gap in the understanding of how AT may influence this parameter. Similarly, flexibility and sleep quality were assessed by only one trial, indicating an urgent need for further research.

### 4.8. Limitations and Strengths

The authors of this review acknowledge some limitations. First, the number of studies that met the selection criteria was limited. However, PRISMA guidelines [27] were followed, six relevant databases were searched, and the grey literature was included. In addition, to ensure the methodological quality of the included studies, the CASPe scale [29] and the Cochrane bias assessment tool [30] were used. Due to the type of intervention, it was not possible for the therapists and participants to remain blinded. However, in nine trials [31,33,34,35,37,38,40,41,43], the assessors remained blinded, ensuring the absence of bias in this regard. On the other hand, there is great heterogeneity in the parameters assessed and the assessment tool used, which impedes the development of a meta-analysis. In addition, there is significant methodological heterogeneity among the studies, particularly in the AT protocols applied (e.g., type of exercises, session duration, frequency, water temperature), which limits the ability to compare results across interventions and draw generalizable conclusions. Finally, this systematic review was registered in PROSPERO (CRD42024528310) to guarantee its originality.

## 5. Conclusions

The efficacy of AT compared to LT for improving balance, gait, and strength in adult PDs appears to be limited. However, preliminary scientific evidence suggests that AT may be particularly superior to LT for improving quality of life, mental health, pain, flexibility, and sleep quality. Ai chi appears to be the most effective therapeutic exercise modality. It has been observed that the practice of supportive therapy does not provide additional benefits when LT is already being performed. No adverse events related to AT have been reported, supporting the fact that AT is a safe treatment strategy. In addition, AT may offer unique physiological advantages, including potential effects on cognitive enhancement, neuroprotection, and inflammation reduction, factors particularly relevant in neurodegenerative diseases. Finally, AT appears to offer benefits in certain outcomes compared to LT, although further studies are needed on biomarkers such as strength and gait, the impact of AT intensity on short- and long-term efficacy, and adherence strategies to aquatic therapy. Moreover, standardizing intervention protocols is essential to improve comparability across studies and to facilitate more robust conclusions in future research.

## Figures and Tables

**Table 1:** Results of methodological quality assessment of included studies of Critical Appraisal Skills Program (CASPe) [29].

Study	Item											Total
	1	2	3	4	5	6	7	8	9	10	11	
Clerici et al. [31] 2019	Yes	Yes	Yes	No	Yes	Yes	B	*p* < 0.05	Yes	Yes	No	9
Kurt et al. [32] 2018	Yes	Yes	Yes	No	Yes	Yes	B	*p* < 0.05	Yes	Yes	Yes	10
Loureiro et al. [40] 2022	Yes	Yes	Yes	No	Yes	Yes	B	*p* < 0.05	Yes	No	Yes	9
Nogueira et al. [41] 2024	Yes	Yes	Yes	No	Yes	Yes	S	*p* < 0.05	Yes	Yes	No	8
Nowak [42] 2018	Yes	Yes	Yes	No	Yes	Yes	M	*p* < 0.05	Yes	Yes	No	9
Palamara et al. [43] 2017	Yes	Yes	Yes	No	Yes	Yes	B	*p* < 0.05	Yes	Yes	No	9
Pérez de la Cruz [35] 2017	Yes	Yes	Yes	No	Yes	Yes	S	*p* < 0.001	Yes	Yes	Yes	9
Pérez de la Cruz [34] 2018	Yes	Yes	Yes	No	Yes	Yes	S	*p* < 0.001	Yes	Yes	Yes	9
Pérez de la Cruz [33] 2019	Yes	Yes	Yes	No	Yes	Yes	S	*p* < 0.001	Yes	Yes	Yes	9
Shahmohammadi et al. [36] 2017	Yes	Yes	Yes	No	Yes	Yes	M	*p* < 0.05	Yes	Yes	Yes	10
Terrens et al. [37] 2020	Yes	Yes	Yes	No	No	Yes	B	*p* < 0.05	Yes	Yes	No	8
Volpe et al. [39] 2014	Yes	Yes	Yes	No	Yes	Yes	B	*p* < 0.05	Yes	Yes	Yes	10
Volpe et al. [38] 2016	Yes	Yes	Yes	No	Yes	Yes	M	*p* < 0.05	Yes	Yes	Yes	10

CASPe questionnaire items. 1: clearly defined question; 2: random assignment; 3: patients considered until the end; 4: blinding; 5: similar groups at baseline; 6: equally treated groups; 7: longer treatment effect; 8: accuracy of effect; 9: applicability to your setting or local population; 10: all outcomes considered; 11: benefits justify risk and cost. Abbreviations. B: big; M: medium; S: small.

**Table 2:** Summary of studies included in the systematic review—participants, intervention characteristics, outcomes, and results.

First Author, Year, and Country of Publication	Study Desing	Participants (Baseline Sample Side and Characteristics)	Intervention	Outcomes	Results
Clerici et al. [31], 2019 Italy	RCT	n_i_ = 60 (8 dropouts → n_f_ = 52); 13 ♀ and 39 ♂. 2–3 H&Y stage. Medication ON IG: n_i_ = 30 (3 dropouts → n_f_ = 27); 8 ♀ and 19 ♂. Age (mean ± SD) = 67 ± 8 years CG: n_i_ = 30 (5 dropouts → n_f_ = 25); 5 ♀ and 20 ♂. Age (mean ± SD) = 67 ± 11 years	IG: 4-week aquatic exercise program and intensive multidisciplinary rehabilitation program. CG: 4-week intensive multidisciplinary rehabilitation program.	Balance: BBS Gait: TUG and FOGQ Aerobic capacity: 6MWT QoL: UPDRS II and UPDRS III	IG: Changes from baseline (d = 0.8) BBS: MD = 7.33 points; *p* < 0.0001 TUG: MD = −3.88 s; *p* < 0.0001 FOGQ: MD = −5.48 points; *p* < 0.0001 6MWT: MD = 86 m; *p* < 0.0001 UPDRS II: MD = −4.85 points; *p* < 0.0001 UPDRS III: MD = −6.26 points; *p* < 0.0001 IG vs. CG (d = 0.8) BBS: MD = −0.23 points; *p* = 0.88 TUG: MD = −0.63 s; *p* = 0.57 FOGQ: MD = 0.36 points; *p* = 0.58 6MWT: MD = 22 m; *p* = 0.19 UPDRS II: MD = −0.49 points; *p* = 0.41 UPDRS III: MD = −0.58 points; *p* = 0.42
Kurt et al. [32] 2018 Turkey	RCT	n_i_ = 40 (0 dropouts → n_f_ = 40); 16 ♀ and 24 ♂. 2–3 H&Y stage. Medication ON IG: n_i_ = 20 (0 dropouts → n_f_ = 20); 9 ♀ and 11 ♂. Age (mean ± SD) = 62.41 ± 6.76 years GC: n_i_ = 20 (0 dropouts → n_f_ = 20); 7 ♀ and 13 ♂. Age (mean ± SD) = 63.61 ± 7.18 years	IG: 5-week Ai Chi program. CG: 5-week land-based exercise program.	Balance: Biodex (API, MLI, OBI) and BBS Gait: TUG QoL: PDQ-39 and UPDRS-III	IG: Changes from baseline (d = 0.8) API: MD = −0.5; *p* < 0.001 MLI: MD = −0.3; *p* < 0.001 OBI: MD = −0.5; *p* < 0.001 BBS: MD = 4.41 points; *p* < 0.001 TUG: MD = −5.01 s; *p* < 0.001 PDQ-39: MD = −4 points; *p* < 0.001 UPDRS-III: MD = −3.29 points; *p* < 0.001 IG vs. CG (d = 0.8) API: MD = −0.4; *p* < 0.001 MLI: MD = −0.15; *p* < 0.001 OBI: MD = −0.62; *p* < 0.001 BBS: MD = 2.5 points; *p* < 0.001 TUG: MD = −3.96 s; *p* < 0.001 PDQ-39: MD = −3 points; *p* < 0.001 UPDRS-III: MD = −1.41 points; *p* < 0.001
Loureiro et al. [40] 2022 Brazil	RCT	n_i_ = 35 (7 dropouts → n_f_ = 28); 12 ♀ and 16 ♂. 1–3 H&Y stage. Medication ON IG: n_i_ = 18 (4 dropouts → n_f_ = 14); 6 ♀ and 8 ♂. Age (median (IQR)) = 69.0 (11.0) years CG: n_i_ = 17 (3 dropouts → n_f_ = 14); 6 ♀ and 8 ♂. Age (median (IQR)) = 63.0 (5.8) years	IG: 9-week WATSU program and land-based exercise program. CG: 9-week land-based exercise program.	QoL: NHP Sleep quality: PSQI	IG: Changes from baseline NHP: MD = 12 points; *p* = 0.001; d = 0.87 PDQI: MD = 6 points; *p* = 0.001; d = 0.85 IG vs. CG NHP: MD = 13 points; *p* < 0.001; d = 0.68 PSQI: MD = 5.5 points; *p* < 0.01; d = 0.78
Nogueira et al. [41] 2024 Brazil and Ireland	RCT	n_i_ = 94 (11 dropouts → n_f_ = 83); 33 ♀ and 50 ♂. 1–3 H&Y stage. Medication ON IG: n_i_ = 22 (1 dropout → n_f_ = 21); 4 ♀ and 17 ♂. Age (mean ± SD) = 66.76 ± 8.97 years CG: n_i_ = 37 (6 dropout → n_f_ = 31); 8 ♀ and 23 ♂. Age (mean ± SD) = 67.87 ± 11.20 years	IG: 12-week aquatic exercise program. CG: 12-week Nordic walking program.	Falls: FES Gait: TUG Aerobic capacity: 6MWT Strength: STS and manual dynamometry QoL: PDQ-39 and UPDRS III	IG: Changes from baseline FES: MD = 2.19 points; *p* > 0.05; d = 0.23 TUG: MD = 0.46 s; *p* > 0.05; d = 0.07 6MWT: MD = 22.37 m; *p* > 0.05; d = 0.24 STS: MD = −1.27 s; *p* > 0.05; d = 0.16 Manual dynamometry: MD = −0.81 kg; *p* > 0.05; d = 0.04 PDQ-39: MD = 0.86 points; *p* > 0.05; d = 0.05 UPDRS III: MD = 1.53 points; *p* > 0.05; d = 0.25 IG vs. CG FES: MD = 0.06 points TUG: MD = 0.16 s 6MWT: MD = −3.41 m STS: MD = −2.36 s Manual dynamometry: MD = 0.43 kg PDQ-39: MD = 4.96 points UPDRS III: MD = 1.04 points
Nowak [42] 2018 South Africa	RCT	n_i_ = 43 (8 dropouts → n_f_ = 35). 1–3 H&Y stage. Medication ON Age (mean ± SD) = 65.2 ± 9.85 years IG: n_i_ = 23 (6 dropouts → n_f_ = 17) CG: n_i_ = 20 (2 dropouts → n_f_ = 18)	IG: 12-week aquatic exercise program. CG: 12-week land-based exercise program.	Posture: kyphosis Balance: BBS Gait: TUG and speed 10 m Strength: STS, knee and ankle isometric, manual dynamometry, knee isokinetic (quadriceps-hamstring relation) Flexibility: SLRT and shoulder ROM Mental health: MHC, BDI and MBCBA. QoL: UPDRS	IG: Changes from baseline Kyphosis: MD = 1; *p* > 0.05 BBS: MD = 2 points; *p* = 0.003 TUG: MD = 1 s; *p* < 0.001 Speed 10 m: MD = 0.3 m/s; *p* < 0.001 STS: MD = 2 reps; *p* < 0.001 Knee isometric: MD = 1.3 kg; *p* = 0.091 Ankle isometric: MD = 2.6 kg; *p* = 0.004 Manual dynamometry: MD = 2.5 kg; *p* = 0.021 Knee isokinetic: MD = 8%; *p* = 0.036 SLRT: MD = 7°; *p* < 0.001 Shoulder ROM: *p* > 0.05 flexion and extension MHC: *p* > 0.05 in all sections BDI: MD = −4 points; *p* = 0.003 MBCBA: *p* > 0.05 in all sections UPDRS: MD = −13 points; *p* < 0.001 IG vs. CG Kyphosis: MD = −4; *p* > 0.05 BBS: MD = 0 points; *p* = 0.352 TUG: MD = 0 s; *p* = 0.998 Speed 10 m: MD = 0 m/s; *p* = 0.999 STS: MD = −1 reps; *p* = 0.971 Knee isometric: MD = −0.1 kg; *p* = 0.116 Ankle isometric: MD = −0.4 kg; *p* = 0.663 Manual dynamometry: MD = 2.9 kg; *p* = 0.603 Knee isokinetic: MD = 1%; *p* = 0.363 SLRT: MD = 3°; *p* = 0.015 Shoulder ROM: *p* > 0.05 flexion and extension MHC: *p* > 0.05 in all sections BDI: MD = −1 points; *p* = 0.771 MBCBA: *p* > 0.05 in all sections UPDRS: MD = 0; *p* = 0.629
Palamara et al. [43] 2017, Italy	RCT	n_i_ = 34 (0 dropouts → n_f_ = 34); 14 ♀ and 20 ♂. 2,5–3 H&Y stage. Medication ON IG: n_i_ = 17 (0 dropouts → n_f_ = 17); 8 ♀ and 9 ♂. Age (mean ± SD) = 70.9 ± 5.7 years CG: n_i_ = 17 (0 dropouts → n_f_ = 17); 6 ♀ and 11 ♂. Age (mean ± SD) = 70.8 ± 5.3 years	IG: 4-week aquatic exercise program and intensive, multidisciplinary rehabilitation program. CG: 4-week intensive multidisciplinary rehabilitation program.	Balance: BBS Gait: TUG QoL: UPDRS II and UPDRS III	IG: Changes from baseline (d = 0.8) BBS: MD = 7.8 points; *p* = 0.0001 TUG: MD = −3.45 s; *p* = 0.001 UPDRS II: MD = −5.1 points; *p* = 0.0005 UPDRS III: MD = −6 points; *p* = 0.0009 IG vs. CG (d = 0.8) BBS: MD = 0.5 points; *p* = 0.99 TUG: MD = −1 s; *p* = 0.99 UPDRS II: MD = 1 points; *p* = 0.88 UPDRS III: MD = 1 points; *p* = 0.99
Pérez de la Cruz [35] 2017 Spain	RCT	n_i_ = 30 (0 dropouts → n_f_ = 30); 16 ♀ and 14 ♂. 1–3 H&Y stage. Medication OFF IG: n_i_ = 15 (0 dropouts → n_f_ = 15). Age (mean ± SD) = 66.80 ± 5.26 years CG: n_i_ = 15 (0 dropouts → n_f_ = 15). Age (mean ± SD) = 67.53 ± 9.89 years	IG: 10-week Ai Chi program. CG: 10-week land-based exercise program.	Pain: VAS Balance: BBS Gait: TUG and Tinetti Strength: STS QoL: UPDRS	IG: Changes from baseline VAS: MD = −1.4 points; *p* < 0.001; d = 0.487 BBS: MD = 4.1 points; *p* < 0.001; d = 0.412 TUG: MD = −2.5 s; *p* < 0.001; d = 0.295 Tinetti: MD = 2.6 points; *p* < 0.001; d = 0.314 STS: MD = −1.7 s; *p* < 0.001; d = 0.225 UPDRS: MD = 0 points; *p* < 0.001; d = 0.516 IG vs. CG VAS: MD = −0.9 points; *p* = 0.005; d = 0.233 BBS: MD = 4.1 points; *p* < 0.001; d = 0.412 TUG: MD = −2.5 s; *p* < 0.001; d = 0.295 Tinetti: MD = 2.9 points; *p* < 0.001; d = 0.418 STS: MD = −1.6 s; *p* = 0.006; d = 0.177 UPDRS: MD = 0.4 points; *p* < 0.001; d = 0.453
Pérez de la Cruz [34] 2018 Spain	RCT	n_i_ = 29 (0 dropouts → n_f_ = 29); 17 ♀ and 12 ♂. 1–3 H&Y stage. Medication OFF IG: n_i_ = 14 (0 dropouts → n_f_ = 14); 9 ♀ and 5 ♂. Age (mean ± SD) = 65.87 ± 7.09 years CG: n_i_ = 15 (0 dropouts → n_f_ = 15); 8 ♀ and 7 ♂. Age (mean ± SD) = 66.44 ± 5.72 years	IG: 11-week Ai Chi program. CG: 11-week land-based exercise program.	Pain: VAS Balance: monopodal balance Gait: TUG Strength: STS Mental Health: GDS QoL: PDQ-39	IG: Changes from baseline VAS: MD = −1.4 points; *p* < 0.001; d = 0.489 Right monopodal balance; MD = 4.2 s; *p* < 0.001; d = 0.495 Left monopodal balance: MD = 2.94 s; *p* < 0.001; d = 0.392 TUG: MD = −2 s; *p* < 0.001; d = 0.284 STS: MD = −1.6 s; *p* = 0.001; d = 0.233 GDS: MD = −0.14 points; *p* = 0.001; d = 0.279 PDQ-39: *p* > 0.05 in all sections except social support IG vs. CG VAS: MD = −1 point; *p* = 0.005; d = 0.248 Right monopodal balance: MD = 4.27 s; *p* < 0.001; d = 0.516 Left monopodal balance: MD = 3 s; *p* < 0.001; d = 0.390 TUG: MD = −1.8 s; *p* < 0.001; d = 0.288 STS: MD = −1.4 s; *p* = 0.006; d = 0.186 GDS: MD = 0.06 points; *p* = 0.002; d = 0.240 PDQ-39: *p* > 0.05 in all sections except social support
Pérez de la Cruz [33] 2019 Spain	RCT	n_i_ = 30 (0 dropouts → n_f_ = 30); 15 ♀ and 15 ♂. 1–3 H&Y stage. Medication OFF IG: n_i_ = 15 (0 dropouts → n_f_ = 15); 8 ♀ and 7 ♂. Age (mean ± SD) = 64.40 ± 5.17 years CG: n_i_ = 15 (0 dropouts → n_f_ = 15); 7 ♀ and 8 ♂. Age (mean ± SD) = 65.83 ± 8.92 years	IG: 10-week Ai Chi program. CG: 10-week land-based exercise program.	Pain: VAS Mental health: GDS QoL: SF-36	IG: Changes from baseline VAS: MD = −1.4 points; *p* < 0.001 GDS: MD = −0.14 points; *p* < 0.001 SF-36: *p* ≤ 0.01 in all sections IG vs. CG VAS: MD = −1 points; *p* = 0.005 GDS: MD = 0.06 points; *p* = 0.002 SF-36: *p* ≤ 0.01 in all sections
Shahmohammadi et al. [36] 2017 Iran and United Kingdom	RCT	n_i_ = 22 (2 dropouts → n_f_ = 20); 20 ♂. 2–3 H&Y stage. Medication ON IG: n_i_ = 11 (1 dropouts → n_f_ = 10); 10 ♂. Age (mean ± SD) = 60.50 ± 5.44 years CG: n_i_ = 11 (1 dropouts → n_f_ = 10); 10 ♂. Age (mean ± SD) = 63.20 ± 4.94 years	IG: 8-week aquatic exercise program. CG: 8-week land-based exercise program.	Balance: Postural sway evaluation in a Kistler force plate (sway range, mean speed, sway area, and mean frequency) QoL: PDQL	IG: Changes from baseline (d = 0.65) Sway range: MD = 21.35 mm; *p* = 0.055 Mean speed: MD = −6.54 mm/s; *p* = 0.001 Sway area: MD = 11.11 mm^2^/s; *p* = 0.001 Mean frequency: MD = −0.11 Hz; *p* = 0.003 PDQL: MD = 21 points; *p* < 0.001 IG vs. CG (d = 0.65) Sway range: MD = 8.9 mm; *p* = 0.52 Mean speed; MD = −3.6 mm/s; *p* = 0.01 Sway area: MD = 12.88 mm^2^/s; *p* = 0.33 Mean frequency: MD = −0.13 HZ; *p* = 0.59 PDQL: MD = 10.8 points; *p* < 0.001
Terrens et al. [37] Australia	RCT	n_i_ = 30 (5 dropouts → n_f_ = 25); 6 ♀ and 24 ♂. 1–3 H&Y stage. Medication ON IG 1: n_i_ = 11 (2 dropouts → n_f_ = 9); 1 ♀ and 10 ♂. Age (mean ± SD) = 74.1 ± 6.6 years IG 2: n_i_ = 10 (1 dropouts → n_f_ = 9); 3 ♀ and 7 ♂. Age (mean ± SD) = 65.6 ± 7.7 years CG: n_i_ = 9 (2 dropouts → n_f_ = 7); 2 ♀ and 7 ♂. Age (mean ± SD) = 76.4 ± 7.4 years	IG 1: 12-week Halliwik program. IG 2: 12-week aquatic exercise program. CG: 12-week land-based exercise program.	Balance: BBS and Mini BESTest. Falls: mFES QoL: UPDRS III	IG 1: Changes from baseline BBS: MedD = 0 points; *p* > 0.05 Mini-best: MedD = 8 points; *p* = 0.011 mFES: MedD = 0.5 points; *p* > 0.05 UPDRS III: MedD = −1 points; *p* > 0.05 IG 1 vs. CG BBS: MedD = −1 points; *p* > 0.05 Mini-Best: MedD = 10 points; *p* > 0.05 mFES: MedD = −1 points; *p* > 0.05 UPDRS III: MedD = 5 points; *p* > 0.05 IG 2: Changes from baseline. BBS: MedD = −1 points; *p* > 0.05 Mini-Best: MedD: −3 points; *p* > 0.05 mFES: MedD: 0.25 points; *p* > 0.05 UPDRS III: MedD: 5 points; *p* > 0.05 IG 2 vs. CG BBS: MedD = 0 points; *p* > 0.05 Mini-best: MedD = −1 points; *p* > 0.05 mFES: MedD = −1.25 points; *p* > 0.05 UPDRS III: MedD = 14 points; *p* > 0.05
Volpe et al. [39] 2914 Italy	RCT	n_i_ = 34 (0 dropouts → n_f_ = 34). 2,5–3 H&Y stage. Medication ON IG: n_i_ = 17 (0 dropouts → n_f_ = 17). Age (mean ± SD) = 68 ± 7 years CG: n_i_ = 17 (0 dropouts → n_f_ = 17). Age (mean ± SD) = 66 ± 8 years	IG: 8-week aquatic exercise program. CG: 8-week land-based exercise program.	Balance: evaluation of the COP sway area with open and closed eyes. BBS and ABC Falls: FES y falls diary. Gait: TUG QoL: PDQ-39, UPDRS II and III	IG: Changes from baseline (d = 0.8) Sway area open eyes: MD = 49.7 mm^2^; *p* = 0.002 Sway area closed eyes: MD = 45.4 mm^2^; *p* = 0.010 BBS: MD = 9.9 points; *p* < 0.0001 ABC: MD = 16.8 points; *p* < 0.0001 FES: MD = −5.9 points; *p* < 0.0001 Falls diary: MD = −2.4; *p* < 0.0001 TUG: MD = −2.0 s; *p* < 0.0001 PDQ-39: MD = −18.4 points; *p* < 0.0001 UPDRS II: MD = −4.3 points; *p* < 0.0001 UPDRS III: MD = −8.3 points; *p* < 0.0001 IG vs. CG (d = 0.8) Sway area open eyes: MD = 24.3 mm^2^; *p* = 0.2871 Sway area closed eyes: MD = 38.5 mm^2^; *p* = 0.0480 BBS: MD = 3.9 points; *p* = 0.0046 ABC: MD = 12.7 points; *p* = 0.0001 FES: MD = −4 points; *p* = 0.0026 Falls diary: MD = −2; *p* = 0.0010 TUG: MD = −0.9 s; *p* = 0.151 PDQ-39: MD = −10.4 points; *p* = 0.0063 UPDRS II: MD = 0.8 points; *p* = 0.4336 UPDRS III: MD = 0.1 points; *p* = 0.9381
Volpe et al. [38] 2016 Italy	RCT	n_i_ = 30 (6 dropouts → n_f_ = 24); 11 ♀ and 19 ♂. 1–3 H&Y stage. Medication ON IG: n_i_ = 15 (2 dropouts → n_f_ = 13); 6 ♀ and 9 ♂. Age (mean ± SD) = 70.6 ± 7.8 years CG: n_i_ = 15 (4 dropouts → n_f_ = 11); 5 ♀ and 10 ♂. Age (mean ± SD) = 70 ± 7.8 years	IG: 8-week aquatic exercise program. CG: 8-week land-based exercise program.	Posture: dorsal and cervical BAK and shoulder symmetry Balance: BBS and ABC. Falls: FES Gait: TUG QoL: PDQ-39 and UPDRS III	IG: Changes from baseline Dorsal BAK: MD = −22.5°; *p* = 0.008 Cervical BAK: MD = −62.2°; *p* < 0.001 Shoulder symmetry: MD = −2.3°; *p* = 0.002 BBS: MD = 3.5 points; *p* < 0.001 ABC: MD = 8.1%; *p* = 0.02 FES: MD = −2.3 points; *p* = 0.027 TUG: MD = −1.4 s; *p* = 0.036 PDQ-39: MD = −9,6 points; *p* < 0.001 UPDRS III: MD = −6.1 points; *p* = 0.001 IG vs. CG Dorsal BAK: MD = −16°; *p* = 0.046 Cervical BAK: MD = −66.9°; *p* = 0.024 Shoulder symmetry: MD = −2.6°; *p* = 0.047 BBS: MD = −3.4 points; *p* > 0.05 ABC: MD = 5.7%; *p* > 0.05 FES: MD = −1 points; *p* > 0.05 TUG: MD = 1.8 s; *p* > 0.05 PDQ-39: MD = −5.4 points; *p* = 0.001 UPDRS: MD = −1.1 points; *p* > 0.05

Abbreviations. ♀: Women. ♂: Men. 6MWT: 6-min walk test. ABC: Activities-specific Balance Confidence Scale. API: Anteroposterior Index. BAK: Body Analysis Kapture. BBS: Berg Balance Scale. BDI: Beck Depression Inventory. CG: Control group. COP: Centre of Pressure. d: Effect size. FES: Falls efficacy scale. FOGQ: Freezing of Gait Questionnaire. GDS: Geriatric Depression Scale. H&Y: Hoehn and Yahr Scale. Hz: Hertz. IG: Intervention group. IQR: Interquartile rate. Kg: Kilograms. m: Metres. mm: Milimetres. MBCBA: Montgomery Borgatta Caregivers Burden Assessment. MD: Mean difference. MedD: Median difference. mFES: Modified Falls Efficacy Scale. MHC: Mental Health Continuum. MLI: Mediolateral Idex. n_f_: Final sample size. n_i_: Initial sample size. NHP: Nottingham Health Profile questionnaire. OBI: Overall balance index. PDQ-39: Parkinson’s disease Questionaire-39. PDQL: Parkinson’s Disease Quality of Life. PSQI: Pittsburg Sleep Quality Index. QoL: Quality of life. RCT: Randomized controlled trials. Reps: Repetitions. ROM: Range of motion. s: Seconds. SD: Standard deviation. SLRT: Straight Leg Raise Test. STS: Sit-to-stand test. TUG: Time up and go. UPDRS-III: Unified Parkinson’s Disease Rating Scale-III. VAS: Visual Analog Scale.

**Table 3:** Characteristics of intervention group and control group interventions.

Author and Year	Exercises	Volume and Intensity	Tª (°C)	Frecuency (Days/Week)	Time (Minutes/Session)	Duration (Weeks)	Supervision
Clerici et al. [31] 2019	IG: Warm-up (walking in different directions, with heels, counter-resistance, and with eyes closed); Principal (proprioception, dual-task walking, obstacles and turns); Cool down (walking and stretching). All submerged in water. + CG intervention	70–80% reserve HR	33–34	3 AT + CG (AT replaces session 1)	60	4	Yes
	CG: Session 1 (cardiovascular warm-up, stretching, ROM work, and core and posture work). Session 2 (work on gait, balance, endurance, and motor control). Session 3 (autonomy in ADL). Session 4 (speech therapy). Day 6 (training with devices).	70–80% reserve HR	-	5 (4 daily sessions) + 1 session with devices	60	4	Yes
Kurt et al. [32] 2018	IG: Warm-up (mobility exercises); Principal (Ai Chi, 16 exercises with slow movements and deep breathing to work on balance, strength, flexibility, and breathing). Cool-down (walking and stretching). All submerged in water.	Not specified	32	5	60	5	Yes
	CG: Warm-up (light aerobic exercise); Principal (stretching, gait, and balance work); Cool-down (slow walking and breathing exercises).	Not specified	-	5	60	5	Yes
Loureiro et al. [40] 2022	IG: Warm-up (recreational activities in the pool); Principal (WATSU, 12 exercises mainly in supine position); Cool-down (massage therapy in pool) + CG intervention	Not specified	34.4 - 36	2 WATSU + 2 land-based	30	9	Yes
	CG: Warm-up (mobility exercises); Principal (exercises with a wide ROM, postural control, and balance); Cool-down (stretching).	Not specified	-	2	30	9	Yes
Nogueira et al. [41] 2024	IG: Warm-up (mobility exercises and stretching); Principal (running, strength exercises, balance, postural control, coordination, dual task, and diving); Cool-down (stretching and relaxation). All submerged in water.	Individualized	-	2	60	12	Yes
	CG: Warm-up (coordination); Principal (Nordic walking); Cool-down (stretching).	Individualized	-	2	60	12	Yes
Nowak [42] 2018	IG: Warm-up (walking); Principal (gait work, joint mobility, balance, and strength exercises); Cool-down (walking and stretching). All submerged in water.	Not specified	-	2	60	12	Yes
	CG: Warm-up (static bicycle), Principal (gait work, joint mobility, balance, and strength exercises); Cool-down (walking and stretching)	Not specified	-	2	60	12	Yes
Palamara et al. [43] 2017	IG: Warm-up (walking in different directions, with heels, counter-resistance, and with eyes closed); Principal (proprioception, dual-task walking, obstacles, and turns); Cool-down (walking and stretching). All submerged in water. + CG intervention	70–80% reserve HR	-	3 AT + CG (AT replaces session 1)	60	4	Yes
	CG: Session 1 (cardiovascular warm-up, stretching, ROM work, and core and posture work). Session 2 (work on gait, balance, endurance, and motor control). Session 3 (autonomy in ADL). Session 4 (speech therapy). Day 6 (training with devices).	70–80% reserve HR	-	5 (4 daily sessions) + 1 session with devices	60	4	Yes
Pérez de la Cruz [35] 2017	IG: Warm-up (recreational exercises); Principal (Ai Chi, 19 exercises emphasizing reach and postural responses, trunk rotation, bipodal, and monopodal balance); Cool-down.	Not specified	30	2	45	10	Yes
	CG: Warm-up (mobility exercises and walking); Principal (aerobic and strength exercises); Cool-down (functional ADL exercises, balance, proprioception, facial exercises, and stretching)	Not specified	-	2	45	10	Yes
Pérez de la Cruz [34] 2018	IG: Warm-up (recreational exercises); Principal (Ai Chi, 19 exercises); Cool-down.	Not specified	30	2	45	11	Yes
	CG: Warm-up (mobility exercises and walking); Principal (aerobic and strength exercises); Cool-down (functional ADL exercises, balance, proprioception, facial exercises, and stretching)	Not specified	-	2	45	11	Yes
Pérez de la Cruz [33] 2019	IG: Warm-up (recreational exercises); Principal (Ai Chi, 10 exercises “Contemplating”, “Floating”, “Uplifting”, “Enclosing”, “Folding”, “Soothing”, “Gathering”, “Freeing”, “Shifting”, “Accepting”); Cool-down.	Not specified	30	2	45	10	Yes
	CG: Warm-up (mobility exercises and gaiting); Principal (aerobic and strength exercises); Cool down (functional ADL exercises, balance, proprioception, facial exercises, and stretching)	Not specified	-	2	45	10	Yes
Shahmohammadi et al. [36] 2017	IG: Warm-up (walking); Principal (gait work, walking in different directions, with heels, tiptoeing, and throwing a ball in different directions); Cool-down (stretching). All submerged in water.	2 series of 10–20 repetitions	30	3	60	8	Yes
	CG: Warm-up (walking); Principal (gait work, walking in different directions, with heels, tiptoeing, and throwing a ball in different directions); Cool down (stretching).	2 series of 10–20 repetitions	-	3	60	8	Yes
Terrens et al. [37] 2020	IG 1: Warm-up (walking); Principal (Halliwick, balance, trunk mobility, core work, and rotations); Cool-down (stretching)	13–14 Borg Scale	34,7	1	60	12	Yes
	IG 2: Warm-up (walking); Principal (balance, aerobic, and strength exercises); Cool-down (stretching). All submerged in water.	13–14 Borg Scale	34,7	1	60	12	Yes
	CG: Warm-up (walking); Principal (balance, aerobic, and strength exercises); Cool-down (stretching).	13–14 Borg Scale	-	1	60	12	Yes
Volpe et al. [39] 2014	IG: Warm-up (aerobic exercises and stretching); Principal (balance training with external disturbances, functional reach, postural responses, and strength exercises); Cool down. All submerged in water.	Not specified	30	5	60	8	Yes
	CG: Warm-up (aerobic exercises and stretching); Principal (balance training with external disturbances, functional reach, postural responses, and strength exercises); Cool-down.	Not specified	-	5	60	8	Yes
Volpe et al. [38] 2016	IG: Warm-up; Principal (balance training with external disturbances); Cool-down (relax exercises). All submerged in water.	Not specified	-	5	60	8	Yes
	CG: Warm-up (aerobic exercises and stretching); Principal (balance training with external disturbance); Cool-down (relax exercises)	Not specified	-	5	60	8	Yes

Abbreviations: ADL: Activities of daily living. AT: Aquatic therapy. CG: Control group. HR: Heart rate. IG: Intervention group. ROM: Range of movement. Tª: Temperature.

**Table 4:** Results of risk of bias assessment of included studies—Cochrane tool [30].

Study	Item						
	1	2	3	4	5	6	7
Clerici et al. [31] 2019	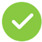	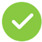	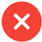	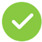	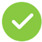	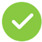	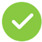
Kurt et al. [32] 2018	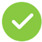	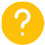	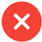	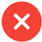	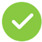	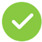	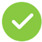
Loureiro et al. [40] 2022	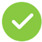	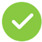	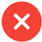	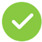	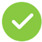	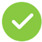	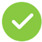
Nogueira et al. [41] 2024	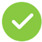	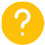	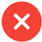	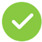	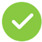	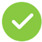	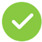
Nowak [42] 2018	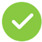	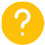	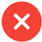	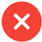	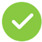	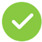	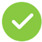
Palamara et al. [43] 2017	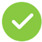	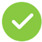	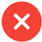	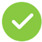	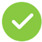	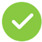	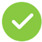
Pérez de la Cruz [35] 2017	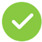	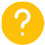	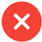	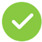	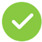	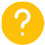	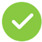
Pérez de la Cruz [34] 2018	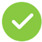	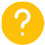	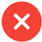	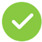	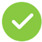	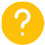	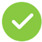
Pérez de la Cruz [33] 2019	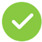	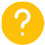	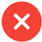	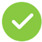	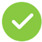	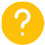	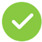
Shahmohammadi et al. [36] 2017	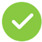	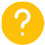	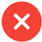	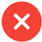	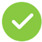	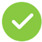	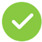
Terrens et al. [37] 2020	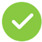	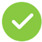	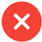	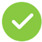	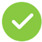	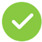	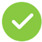
Volpe et al. [39] 2014	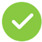	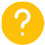	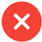	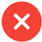	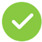	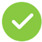	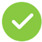
Volpe et al. [38] 2017	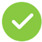	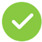	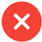	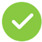	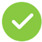	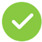	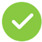

Cochrane tool items. 1: random sequence generation; 2: assignment concealment; 3: blinding of participants and therapist; 4: blinding of assessors; 5: incomplete results data; 6: selective reporting results; 7: other biases. Abbreviations: “✓”: low risk of bias; “X”: high risk of bias”; “?”: uncertainty about the potential for bias or lack of information in this regard.

## Abbreviations

The following abbreviations are used in this manuscript:

AMPK	AMP-activated protein kinase
AT	Aquatic-based therapeutic exercise
BDNF	Brain-derived neurotrophic factor
CASPe	Critical Appraisal Skills Program in Spanish
CG	Control group
FNDCS	Fibronectin type III domain-containing proteins
IG	Intervention group
IGF-1	Insulin-like growth factor 1
IL-10	Interleukin 10
LT	Land-based therapeutic exercise
MeSH	Medical Subject Headings
PD	Parkinson’s Disease
PEDro	Physiotherapy Evidence Database
QoL	Quality of life
TNFα	Tumor necrosis factor-alpha
PGC-1α	Peroxime proliferator-activated receptor gamma coactivator 1-alpha
PRISMA	Preferred Reporting Items for Systematic Reviews and Meta-analyses
Tpa	Tissue plasminogen activator

## Appendix A. Checklist PRISMA 2020 [27]

**Section and Topic**	**Item**	**Checklist Item**	**Location Where Item Is Reported**
Title			
Title	1	Identify the report as a systematic review.	1
Abstract			
Abstract	2	See the PRISMA 2020 for Abstracts checklist.	1–2
Introduction			
Rationale	3	Describe the rationale for this review in the context of existing knowledge.	2
Objectives	4	Provide an explicit statement of the objective(s) or question(s) the review addresses.	2
Methods			
Eligibility criteria	5	Specify the inclusion and exclusion criteria for this review and how studies were grouped for the syntheses.	3
Information sources	6	Specify all databases, registers, websites, organizations, reference lists, and other sources searched or consulted to identify studies. Specify the date when each source was last searched or consulted.	3
Search strategy	7	Present the full search strategies for all databases, registers, and websites, including any filters and limits used.	3 and 29
Selection process	8	Specify the methods used to decide whether a study met the inclusion criteria of this review, including how many reviewers screened each record and each report retrieved, whether they worked independently, and, if applicable, details of automation tools used in the process.	3
Data collection process	9	Specify the methods used to collect data from reports, including how many reviewers collected data from each report, whether they worked independently, any processes for obtaining or confirming data from study investigators, and if applicable, details of automation tools used in the process.	3
Data items	10a	List and define all outcomes for which data were sought. Specify whether all results that were compatible with each outcome domain in each study were sought (e.g., for all measures, time points, analyses), and if not, the methods used to decide which results to collect.	3
	10b	List and define all other variables for which data were sought (e.g., participant and intervention characteristics, funding sources). Describe any assumptions made about any missing or unclear information.	3
Study risk of bias assessment	11	Specify the methods used to assess the risk of bias in the included studies, including details of the tool(s) used, how many reviewers assessed each study, whether they worked independently, and, if applicable, details of automation tools used in this process.	3
Effect measures	12	Specify for each outcome the effect measure(s) (e.g., risk ratio, mean difference) used in the synthesis or presentation of results.	-
Synthesis methods	13a	Describe the processes used to decide which studies were eligible for each synthesis (e.g., tabulating the study intervention characteristics and comparing against the planned groups for each synthesis (item #5)).	-
	13b	Describe any methods required to prepare the data for presentation or synthesis, such as handling missing summary statistics or data conversions.	-
	13c	Describe any methods used to tabulate or visually display results of individual studies and syntheses.	-
	13d	Describe any methods used to synthesize results and provide a rationale for the choice(s). If meta-analysis was performed, describe the model(s) and method(s) to identify the presence and extent of statistical heterogeneity and software package(s) used.	-
	13e	Describe any methods used to explore possible causes of heterogeneity among study results (e.g., subgroup analysis, meta-regression).	-
	13f	Describe any sensitivity analyses conducted to assess robustness of the synthesized results.	-
Reporting bias assessment	14	Describe any methods used to assess risk of bias due to missing results in a synthesis (arising from reporting biases).	-
Certainty assessment	15	Describe any methods used to assess certainty (or confidence) in the body of evidence for an outcome.	-
Results			
Study selection	16a	Describe the results of the search and selection process, from the number of records identified in the search to the number of studies included in this review, ideally using a flow diagram.	3–5
	16b	Cite studies that might appear to meet the inclusion criteria but which were excluded, and explain why they were excluded.	3–5
Study characteristics	17	Cite each included study and present its characteristics.	8 and 10–18
Risk of bias in studies	18	Present assessments of risk of bias for each included study.	6–8
Results of individual studies	19	For all outcomes, present, for each study: (a) summary statistics for each group (where appropriate) and (b) an effect estimate and its precision (e.g., confidence/credible interval), ideally using structured tables or plots.	8–16 and 19
Results of syntheses	20a	For each synthesis, briefly summarize the characteristics and risk of bias among contributing studies.	-
	20b	Present results of all statistical syntheses conducted. If meta-analysis was performed, present for each the summary estimate and its precision (e.g., confidence/credible interval) and measures of statistical heterogeneity. If comparing groups, describe the direction of the effect.	-
	20c	Present results of all investigations of possible causes of heterogeneity among study results.	-
	20d	Present results of all sensitivity analyses conducted to assess the robustness of the synthesized results.	-
Reporting biases	21	Present assessments of risk of bias due to missing results (arising from reporting biases) for each synthesis assessed.	-
Certainty of evidence	22	Present assessments of certainty (or confidence) in the body of evidence for each outcome assessed.	-
Discussion			
Discussion	23a	Provide a general interpretation of the results in the context of other evidence.	19–23
	23b	Discuss any limitations of the evidence included in this review.	23
	23c	Discuss any limitations of the review processes used.	23
	23d	Discuss implications of the results for practice, policy, and future research.	21–23
Other information			
Registration and protocol	24a	Provide registration information for this review, including register name and registration number, or state that this review was not registered.	2
	24b	Indicate where the review protocol can be accessed, or state that a protocol was not prepared.	2
	24c	Describe and explain any amendments to information provided at registration or in the protocol.	2
Support	25	Describe sources of financial or non-financial support for this review and the role of the funders or sponsors in this review.	24
Competing interests	26	Declare any competing interests of review authors.	24
Availability of data, code, and other materials	27	Report which of the following are publicly available and where they can be found: template data collection forms; data extracted from included studies; data used for all analyses; analytic code; any other materials used in this review.	-

## Appendix B. Search Strategy Employed in Databases

**Database**	**Search Strategy**
Medline/Pubmed	(Parkinson Disease (MeSH) OR Parkinson OR Parkinson’s Disease) AND (Aquatic Therapy (MeSH) OR Aquatic Exercise Therapy OR Water Exercise Therapy OR Ai Chi Therapy)
Scopus	(TITLE-ABS-KEY (“parkinson disease” OR Parkinson OR “Parkinson’s Disease”) AND TITLE-ABS-KEY (“Aquatic Therapy” OR “Aquatic Exercise Therapy” OR “Water Exercise Therapy” OR “ai chi therapy”))
Web of Science	Parkinson Disease (topic) AND Aquatic Therapy (Topic)
PEDro	Parkinson disease AND hydrotherapy, balneotherapy
CINAHL	(Parkinson disease OR Parkinson OR Parkinson’s Disease) AND (Aquatic therapy OR Aquatic Exercise Therapy OR Water Exercise Therapy OR ai chi therapy)
Cochrane	Parkinson Disease AND Aquatic Therapy
